# ROS: Basic Concepts, Sources, Cellular Signaling, and its Implications in Aging Pathways

**DOI:** 10.1155/2022/1225578

**Published:** 2022-10-19

**Authors:** Arthur José Pontes Oliveira de Almeida, Júlio César Pinheiro Lúcio de Oliveira, Larisse Virgolino da Silva Pontes, Javanyr Frederico de Souza Júnior, Tays Amanda Felisberto Gonçalves, Sabine Helena Dantas, Mathania Silva de Almeida Feitosa, Antonia Oliveira Silva, Isac Almeida de Medeiros

**Affiliations:** ^1^Departamento de Ciências Farmacêuticas/Centro de Ciências da Saúde, Universidade Federal da Paraíba, Cidade Universitária-Campus I, Caixa Postal 5009, 58051-970 João Pessoa, PB, Brazil; ^2^Programa de Pós-Graduação em Enfermagem/Centro de Ciências da Saúde, Universidade Federal da Paraíba, Cidade Universitária-Campus I, Caixa Postal 5009, 58051-970 João Pessoa, PB, Brazil

## Abstract

Reactive oxygen species (ROS) are bioproducts of cellular metabolism. There is a range of molecules with oxidizing properties known as ROS. Despite those molecules being implied negatively in aging and numerous diseases, their key role in cellular signaling is evident. ROS control several biological processes such as inflammation, proliferation, and cell death. The redox signaling underlying these cellular events is one characteristic of the new generation of scientists aimed at defining the role of ROS in the cellular environment. The control of redox potential, which includes the balance of the sources of ROS and the antioxidant system, implies an important target for understanding the cells' fate derived from redox signaling. In this review, we summarized the chemical, the redox balance, the signaling, and the implications of ROS in biological aging.

## 1. Introduction

All living organisms produce reactive oxygen species (ROS) as a result of cellular metabolism [1, 2]. Despite the presence of antioxidant defenses (e.g., enzymes, proteins, and vitamins) in organisms, ROS can accumulate due to overproduction or failure in the antioxidant system, leading to the stage of “oxidative stress” [3], a key hallmark of aging [4–6].

Harman, in 1956, proposed the “free radical theory of aging,” which argued that ROS derived from metabolism were the primary cause of aging [7]. Since then, several publications have reported the deleterious effects of ROS on aging [8, 9]. However, recent studies on long-lived models and genetically altered animals challenge the role of ROS in aging [10]. In this way, ROS appear to have a dual effect, first as an activator of a homeostatic compensatory response that increases with age to maintain survival, and then, beyond a certain point, as a factor that, rather than alleviating, aggravat the damages associated with aging [11, 12].

In this context, this review drafts an integrated approach to the dual role of ROS on aging, addressing molecular, cellular, and physiological aspects of ROS.

## 2. A General Overview of ROS

A range of molecules with oxidizing properties contributes to oxidative stress [3]. These ROS include superoxide anions (O_2_^• −^), hydroxyl radical (HO^•^), nitric oxide (^•^NO), and lipid radicals, which present unpaired electrons being considered as free radicals [13]. Other ROS that has oxidizing properties but is not free radicals include hydrogen peroxide (H_2_O_2_), peroxynitrite (ONOO¯), and hypochlorous acid (HOCl) [14]. It is important to note that referring to ROS as a group with the same proprieties may imply the erroneous conception that all these chemical species play the same role in the cellular environment. Thereby, events such as signalization or damage might have specific agents. Furthermore, ROS present a dual role depending on different aspects such as cellular environment, time exposure, concentration, and compartmentalization.

The three primary species, i.e., the O_2_^•−^, H_2_O_2_, and HO^•^. They are produced from molecular oxygen during the successive four steps of 1-electron reduction (Reaction (1)). Moreover, many oxidants are derived from these primary sources of ROS.

(1) $$\begin{array}{c} {\text{O}}_{2}\overset{+{\text{e}}^{-}}{\to }{{\text{O}}_{2}}^{•-}\overset{+{\text{e}}^{-}\left(+2{\text{H}}^{+}\right)}{\to }{\text{H}}_{2}{\text{O}}_{2}\overset{+{\text{e}}^{-}}{\to }{\text{HO}}^{•}+{\text{HO}}^{-}\overset{+{\text{e}}^{-}\left(+2{\text{H}}^{+}\right)}{\to }2{\text{H}}_{2}\text{O} \end{array}$$

Hence, O_2_^• −^ is produced enzymatically by the monovalent reduction of oxygen, and its presence is involved with physiological signaling and diseases associated with oxidative stress [15]. Although there are several sources of O_2_^• −^ intracellularly, cellular mechanisms of detoxification balance the levels of O_2_^• −^ through the enzyme superoxide dismutase (SOD), which results in the release of H_2_O_2_ (less toxic) [16]. O_2_^• −^ also reacts with H_2_O_2_ to generate HO^•^ (Haber-Weiss reaction–see below), the most potent oxidant of ROS, and can react with ^•^NO to form ONOO¯ (Reaction (2)), resulting in damaged proteins, lipids, carbohydrates, and DNA. In fact, HO^•^ and ONOO¯ are not considered as signaling molecules due to their highly reactive nature, but they contribute significantly to oxidative stress and tissue damage [17]. In addition, HO^•^ and ONOO¯ cannot be enzymatically removed, being very harmful to the organism.

(2) O2•−+NO •⟶ONOO−

H_2_O_2_ acts as a second messenger, regulating redox signaling at homeostatic physiological levels [18, 19]. Their levels ensure an adaptive response to stress, necessary for cellular survival. However, H_2_O_2_ concentration in the extracellular and intracellular space is crucial for the metabolism and survival of the cells [20]. Extracellular H_2_O_2_ can enter the cells through aquaporin water channels (AQP) [21]. To maintain basal levels of H_2_O_2_, enzymes such as catalase (CAT) and glutathione peroxidases (GPx) break H_2_O_2_ into water and oxygen. On the other hand, in the presence of chloride ions, H_2_O_2_ is converted to HOCl through myeloperoxidases (MPO) (Reaction (3)), which is highly reactive and plays a vital role in oxidative damage, including the mechanism by which the immune system kills pathogens [22].

(3) $$\begin{array}{c} {\text{H}}_{2}{\text{O}}_{2}+{\text{H}}^{+}+{\text{CI}}^{-}\overset{\text{MPO}}{⟶}\text{HOCI}+{\text{H}}_{2}\text{O} \end{array}$$

Radical HO^•^ results from the partial reduction of H_2_O_2_. They are very reactive that have a strong affinity for electron-rich sites such as aromatic or sulfur-containing molecules such as proteins and DNA. Two reactions often related to HO^•^ production are the Fenton reaction and Haber-Weiss reaction (Reaction (4)).

(4) $$\begin{array}{c} {\text{Fe}}^{3+}+{{\text{O}}_{2}}^{•-}⟶{\text{Fe}}^{2+}+{\text{O}}_{2}\\ {\text{Fe}}^{2+}+{\text{H}}_{2}{\text{O}}_{2}⟶{\text{Fe}}^{3+}+{\text{HO}}^{•}+{\text{HO}}^{-} \end{array}$$

Net reaction:

(5) $$\begin{array}{c} {{\text{O}}_{2}}^{•-}+{\text{H}}_{2}{\text{O}}_{2}⟶{\text{HO}}^{•}+{\text{HO}}^{-}+{\text{O}}_{2} \end{array}$$

The Haber-Weiss reaction occurs in two steps, the second step is known as the Fenton reaction. Moreover, high levels of irons (3) and (2) are related to an increase in oxidative stress and, eventually, to massive cell death.

Other oxygen-derived free radicals are the peroxyl radicals (ROO^•^). The simplest form of these radicals is the hydroperoxyl radical (HOO^•^) (Reaction (6)), which plays a role in fatty acid peroxidation [23]. Peroxyl radical starts a chain reaction that converts polyunsaturated fatty acids into lipid hydroperoxides, which are highly unstable and easily decompose to secondary products like aldehydes (like 4-hydroxy-2,3-nonenal) and malondialdehydes (MDAs) [24]. Because they are very reactive, these products are biomarkers of oxidative stress, which lead to protein and DNA damage, triggering the loss or alterations in their functions [25].

(6) $$\begin{array}{c} {\text{Fe}}^{3+}+{\text{H}}_{2}{\text{O}}_{2}⟶{\text{Fe}}^{2+}+{\text{HOO}}^{•}+{\text{H}}^{+} \end{array}$$

^•^NO is a pleiotropic regulator and effector of the immune, cardiovascular, and nervous systems [26]. When at optimal levels, ^•^NO has antiplatelet, antithrombotic, and anti-inflammatory functions, which explains ^•^NO's benefits in those systems [27, 28]. However, ^•^NO may have adverse effects via pro-oxidant pathways despite its benefits. Indeed, ^•^NO can form other radicals with potent oxidative properties, such as nitrogen dioxide (^•^NO_2_) (Reaction (7)) and ONOO¯ [29]. ONOO¯ can react with CO_2_ to generate nitrosoperoxycarbonate (ONOOCO_2_¯), and eventually carbonate ion (CO_3_^• -^) (reaction (8)). Moreover, ^•^NO can be converted in nitrite and nitrate (Reaction (10)), which aggravates the RNS toxicity.

(7) ONOO−⟶H+ONOOH⟶HO•+NO •2

(8) ONOO−+CO2⟶ONOOCO2−⟶CO3•−+NO •2

Thereby, RNS play a key role in healthy such (e.g., ^•^NO) and diseases (e.g., ONOO¯). Indeed, high levels of ONOO¯ and depletion of ^•^NO levels are related to endothelial dysfunction, an event present in aging and in the genesis of several age-related diseases such as the cardiovascular diseases (CVDs) [4].

## 3. Intracellular Sources of ROS

**Figure 1:**
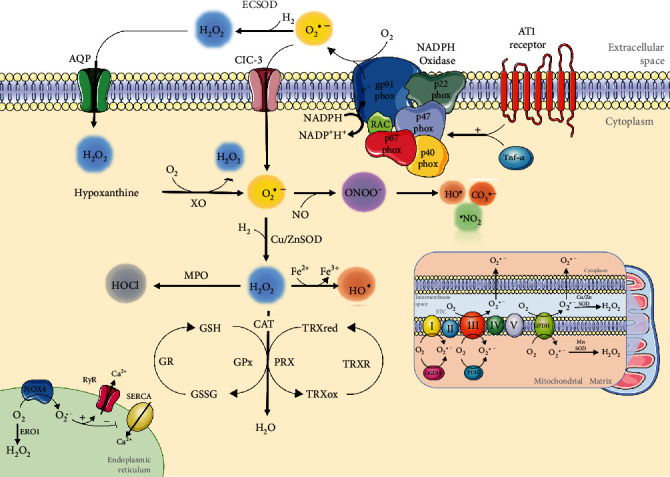
Schematic overview of ROS production/elimination. NOX, located in the plasma membrane, produces O_2_^• −^ in the extracellular space by transferring an electron from cytoplasmic NADPH to O_2_. O_2_^• −^ can be targeted by the ECSOD enzyme and converted into H_2_O_2_, which can permeate the plasma membrane by aquaporins or be transported to the intracellular space by ClC-3. In the cytoplasm, O_2_^• −^ can be produced by XO. In addition, O_2_^• −^ reacts with ^•^NO to form ONOO^−^, whose decomposition results in the formation of some very reactive species, such as ^•^OH, ^•^NO_2_, and CO_3_^• -^. However, the cytoplasmic isoform Cu/ZnSOD can act in O_2_^• −^, producing H_2_O_2_ targeted by MPO, forming HOCl, or by CAT, GPx, and peroxiredoxins (PRX), forming H_2_O. However, through the Fenton reaction, H_2_O_2_ is reduced to ^•^OH, a highly toxic radical in the presence of iron. In the mitochondria, electron transport chain (ETC) complexes I and III are the main sites of oxidant production, with O_2_^• −^ production occurring both on the mitochondrial matrix side and in the intermembranous space of the mitochondria. Other important sources of ROS include the endoplasmic reticulum (ER), which impacts calcium signaling and proteostasis directly. Abbreviations: NADPH oxidase (NOX); chloride channel-3 (ClC-3); xanthine oxidase (XO); myeloperoxidase (MPO); endoplasmic reticulum oxidoreductin 1 (ERO1); ryanodine receptors (RyRs); sarco/endoplasmic reticulum Ca^2+^-ATPase (SERCA); oxoglutarate dehydrogenase (OGDH); pyruvate dehydrogenase complex (PDH); tumour necrosis factor *α* (Tnf-*α*); glutathione reduced (GSH); glutathione oxidized (GSSG); glutathione reductase (GR); thioredoxin (TRX); and thioredoxin reductase (TRXR).

There are several intracellular sources of ROS such as the mitochondria and NADPH oxidases (NOX), the major sources of intracellular ROS [30]. Interestingly, both sources have interactions between them, which contribute to the gradual progression of oxidative stress [31]. Other sources of ROS include uncoupled NO synthase, cytochrome p450, xanthine oxidase (XO), the endoplasmic reticulum, peroxidases, and cyclooxygenases [32, 33] (Figure 1).

### 3.1. Mitochondrial Dysfunction

The mitochondria are the primary source of ROS during aging [34]. After discovering that in aerobic conditions, mitochondria use oxygen oxidation to produce ATP, [35] reviewed his “free radical theory of aging,” indicating that when mitochondria are deficient, it results in high production of O_2_^• −^, raising mitochondrial ROS (mtROS) levels, which lead to the accumulation of mitochondrial DNA (mtDNA) mutations, resulting in aging “mitochondrial theory of aging” [35]. However, recent evidence involving mtROS using longevity animal models, on the other hand, rejects, at least in part, the initial premise of the mitochondrial theory of aging [36]. Furthermore, while the role of mtROS in aging remains unclear, it is clear that mtROS play a role in the development of age-related diseases [37].

The electron transport chain (ETC) is responsible for massive production of O_2_^• −^ [38]. ETC comprises five complexes (complex I to V) of proteins responsible for ATP generation [39]. The NADPH-ubiquinone oxidoreductase complex (Complex I) is considered the main site for mtROS production, and external factors, such as hypoxia, exacerbate this phenomenon [40]. In complex I, superoxide anion can be formed in the hydrophilic domain of the enzyme when one of the electrons in NADH_2_ that is transferred to the flavin mononucleotide (FMN) overflows and is transferred to O_2_ and also when reduced flavin mononucleotide (FMNH_2_) transfers an electron to O_2_ [41]. Complex III (ubiquinol-cytochrome c oxidoreductase) accepts reducing equivalents formed in complexes I and II and processes them by the Q-cycle operating mechanism. Overproduction of ROS by complex III may result from acquired and genetic defects in the mitochondrial respiratory chain. Therefore, the potential role of complex III as a cause of gross mitochondrial ROS production under the steady-state physiological mode of operation remains unclear. Furthermore, the enzymes glycerol-3-phosphate dehydrogenase, 2-oxoglutarate, pyruvate dehydrogenase, and electron transfer flavoprotein Q oxidoreductase (ETFQOR) are essential sources of mtROS [42]. The mitochondrial damage alters its function, decreasing ATP production and increasing ROS levels, resulting in a positive feedback process that amplifies its damage [43]. In contrast, uncoupling proteins (UCPs) decrease the membrane potential of mitochondria in oxidative phosphorylation, which attenuates ROS production and promotes cytoprotection, including in the aging process [44]. UCPs are transport proteins of the inner mitochondrial membrane responsible for inducing proton leakage and dissipating the protonmotive force (*Δ*p) [45]. Five UCPs (UCP1-5) have been discovered in mammals [46], in which UCP2 and UCP3 act to protect against mitochondrial oxidative damage [47]. UCP2 and UCP3 dissipate the proton gradient to prevent *Δ*p turn excessive, thus decreasing the ROS produced by electron transport [48]. However, UCP2 still acts as a mitochondrial oxidative stress sensor, and its function is a vital component of the local feedback mechanisms that control mitochondrial ROS production [48]. Furthermore, swallowing during oxidative stress can activate proton leakage through UCP2, which is believed to be implicated in reduced superoxide production [45].

Inflammatory cytokines are associated with increased production of ROS by mitochondria in stress conditions [49]. For example, Tnf-*α* can decrease the regular activity of complex I, leading to mitochondrial dysfunction and cell death [50]. Moreover, it has been shown that mitochondrial dysfunction increases cellular sensitivity to several proinflammatory cytokines and active cellular inflammatory pathways, which can, in the end, amplify cellular damage and ROS production [51]. Damaged mitochondria also release proapoptotic factors, such as cytochrome C and secretory associated senescence phenotype (SASP), an essential organelle related to cell aging and age-related diseases [52].

### 3.2. NADPH Oxidases

NADPH oxidases (NOX) are an essential source of ROS, especially in the cardiovascular system [53]. Excessive production of ROS mediated by NOX contributes to cardiovascular pathogenesis [54]. In addition, overexpression or high activity of NOX are implicated in numerous age-related diseases [55, 56]. NOX1, NOX2, NOX3, NOX4, NOX5, Duox1, and Duox2 are the seven isoforms of NOX identified, which differ in their location, activation, and type of ROS that they produce [57].

All of these isoforms of the NOX family are transmembrane proteins and act by transporting electrons through biological membranes to reduce oxygen in superoxide anions (NOX1-3 and NOX5) or hydrogen peroxide (NOX4) [58]. ROS formation initially occurs with the binding of NADPH to the dehydrogenase domain, and the electrons are transferred sequentially from the NADPH substrate to the FAD cofactor and then to the two heme groups in the transmembrane domain. On the other side of the membrane, the final electron acceptor is oxygen, reduced to form superoxide or hydrogen peroxide [58]. A variety of factors can stimulate ROS production through NADPH oxidase, Toll-like receptor agonists, leptin, cytokines (IL-6, PDGF, TGF-*β*, and Tnf-*α*) and hormones such as angiotensin II (Ang II), aldosterone, and endothelin-1 (ET-1) [59].

Three isoforms have been related to senescence induction: NOX1, NOX2, and NOX4 [60]. Sfeir et al. showed that senescent endothelial cells present NOX1 and NOX4 overexpression [61]. In addition, there was an increase in the expression of NOX4 in the aorta of aged rats compared to young rats, which was associated with vascular impairment and hypertrophy. NOX4 knockout rats, on the other hand, had none of these characteristics [62]. Corroborating these results, accumulated evidence has shown that NOX4 is a critical regulatory gene for expressing inflammatory mediators in vascular smooth muscle cells (VSMC) in aging, being a critical factor for the development of CVDs age-related atherosclerotic severity [63]. NOX regulation, which affects ROS levels, plays a crucial role in developing aging and age-related diseases.

### 3.3. Xanthine Oxidase

The final two steps of purine metabolism in humans are catalyzed by xanthine oxidase (XO), which catalyzes the oxidative hydroxylation of hypoxanthine to xanthine and xanthine to uric acid [64]. O_2_ quickly accepts electrons derived from hypoxanthine and xanthine oxidation by XO to generate O_2_^• −^ and H_2_O_2_ [65]. XO is upregulated in aging, which is associated with oxidative stress, immunosenescence, and inflammation [66]. In addition, XO seems to be a marker of endothelial dysfunction, a critical process leading to several age-related diseases, such as CVDs [67, 68]. On the other hand, XO inhibitors such as allopurinol, prevent oxidative stress, atrial remodeling [69], fibrosis, and myocardial injuries in aged rats [70].

### 3.4. Uncoupled NO Synthase

In mammals, NO can be generated by three different isoforms of the enzyme NO synthase (NOS). The isozymes are referred to as neuronal nNOS (or NOS I), inducible form iNOS (or NOS II), and endothelial eNOS (or NOS III) [71]. In aging, eNOS (a biomarker of endothelial health) are dysfunctional, primarily by losing its catalytic activity, resulting in eNOS uncoupling [28]. Several mechanisms are proposed to play a role in this endothelial event, such as the depletion of eNOS cofactor BH_4_, L-arginine deficiency, and increase of endogenous eNOS inhibitor, asymmetric dimethylarginine (ADMA) [72]. Uncoupled eNOS produces superoxide, which scavenges NO to form ONOO¯, a short-lived and powerful oxidant promoting oxidation and nitration reactions that affect different biomolecules, including lipids, proteins, and DNA [73]. In addition, ONOO¯ oxidizes BH_4_, an eNOS cofactor, to BH_3_ and subsequently to BH_2_, which binds with high affinity to eNOS without supporting its catalytic activity [74]. ONOO¯ also reduces endothelial transport of L-arginine, the exclusive substrate for eNOS, and increases the rate of L-arginine efflux [75]. Moreover, ONOO¯ directly oxidizes the reduced glutathione (GSH), its endogenous scavenger, which plays a significant role in the cellular defense against ROS. Similarly, via nitration of SOD, ONOO¯ inactivates this enzyme, leading to diminished antioxidant cellular defense mechanisms and increased superoxide levels [76].

### 3.5. Endoplasmic Reticulum

In eukaryotic cells, the endoplasmic reticulum (ER) is responsible for cellular ROS production, being the ER oxirreductin-1 (ERO1) is the primary source of these ROS [77]. In healthy conditions, an optimal level of ROS is essential for forming disulfide bonds, a critical process in the protein folding machinery [78]. Hence, the protein folding success in the ER depends on the appropriate environment in the organelle, such as redox potential and metabolic needs [79]. In contrast, ER dysfunction can generate unfolded and misfolded proteins, activating the Unfolded Protein Response (UPR), which is fundamental for the resolution of protein malfunction [80].

The inositol-requiring kinase 1 (IRE1), a transmembrane protein, is the leading and most conserved ER stress sensor, with the Ser/Thr kinase domain and an RNAase domain in the cytosolic portion [81]. The RNAase function of IRE1 is related to inflammatory signaling pathways of the inflammasome NRLP3 through the cleavage of microRNAs [82]. Moreover, the kinase portion of IRE1 is capable of activating the adaptive tumor protein tumor necrosis *α* (Tnf-*α*) receptor-associated factor 2 (TRAF2) and, subsequently, the NF*κ*B pathways and c-Jun N-terminal kinase (JNK) pathway [83]. These pathways are directly associated with increasing and maintaining ROS cell production [84].

Moreover, the release of Ca^2+^ by ER can trigger ROS production [85]. The redox-sensing thiol groups control the Ryanodine Receptor (RyR) (Ca^2+^ channel present in the ER). The oxidation of RyR thiols sites can activate RyR, allowing the release of calcium by the ER [86]. Deregulated calcium signaling is closely related to mitochondrial dysfunction and NADPH oxidase hyperactivity, prominent cellular sources of ROS [87]. ER also expresses one of the isoforms of NADPH oxidase, NOX4, which contributes to the vicious cycle of oxidative stress in the ER [62].

### 3.6. Cytochrome p450

Cytochromes p450 (CYP) are monooxygenase enzymes metabolically active in phase I first-pass reactions in the metabolism of drugs, dietary chemicals, and endogenous molecules [88]. The CYP superfamily consists of 57 genes classified into 18 families and 44 subfamilies [89]. Four components form the CYP catalytic system: the substrate, a P450 enzyme (an enzyme that performs oxidative catalysis), a redox agent (performs electron transfer—NADPH cytochrome P450 reductase, and cytochrome b5), and the NADPH cofactor (provides reducing equivalents) [90].

CYP-catalyzed monooxygenation reactions occur by binding this enzyme to its substrate utilizing an oxygen molecule to form the oxi complex [91]. The oxi complex is reduced to a peroxide complex via NADPH electron transfer, which is rapidly protonated by releasing a water molecule and forming a highly reactive intermediate compound, which captures adjacent hydrogen atoms in the substrate to produce ROS. After this free radical formation, the substrate is released from the active site, and the enzyme returns to its resting state [89]. Although, as in this redox system, the stereometric molar relationship is not achieved between oxygen consumption and hydroxylated products, there is an excess in ROS formation through the release of superoxide anions and hydrogen peroxide. Thus, xenobiotic metabolism also plays an important role in cytotoxicity induced by ROS formation [92].

However, the aging process is associated with a decline in liver function, leading to changes in biotransformation [93]. Many CYP genes, especially from CYP 1-3 families, show a reduction in expression in older rats [94]. In addition, there is a reduction in the mRNA of hepatic CYP with aging [95]. Furthermore, the ability of liver CYP to metabolize xenobiotics decreases with aging [96]. Therefore, the sustained increase in ROS in the aging process seems to come from other sources.

### 3.7. Peroxidases

Peroxidases consist of a class of enzymes responsible for removing cellular peroxide radicals [97]. In mammals, the prototypes are catalase and glutathione peroxidases (GPx) [97]. Among its functions, we highlight the removal of peroxides produced by cell machinery and those originated by the biotransformation of xenobiotics (such as ethanol) [98]. In this context, these two types of peroxidases oppose cellular oxidative stress [99].

However, haloperoxidases, another group of peroxidases, mediate reactions of halide ions with hydrogen peroxide [100]. This class has identified three enzymes in mammals: lactoperoxidase, myeloperoxidase (MPO), and eosinophil peroxidase. These enzymes can oxidize iodide, bromide, and chloride ions to form reactive halogen species such as HOCl [101], responsible for enhancing the immunity clearance of several pathogens [102]. On the other hand, it contributes to oxidative stress when it is persistent and eventually leads to protein, lipid, and DNA damage [103].

### 3.8. Cyclooxygenases

The enzyme cyclooxygenase (COX) is responsible for the metabolism of arachidonic acid by generating prostaglandin precursors, which contribute to inflammation, ROS production, and lipid oxygenation [104]. COX has two isoforms, COX-1 and COX-2. COX-1 is a ubiquitous isoform constitutively expressed in all tissues, participating in physiological actions. COX-2 is expressed in various tissues, including the kidney, gastrointestinal tract, brain, lungs, and thymus, and its synthesis can be induced in various tissues during inflammatory processes [105]. COX-2 expression is transcriptionally transcribed by Nf-*κ*B, AP-1, and CREB, which can act synergistically or independently on this enzyme's gene expression. In addition, increased ROS production also plays an essential role in the positive regulation of COX-2 [106]. This event is triggered in response to increased NADPH oxidase activity, leading to activation of Akt and MAPK cascades, which promote COX-2 expression [107]. COX-2-mediated proinflammatory signs in cellular senescence are also activated in response to Nf-*κ*B and contribute to controlling mitochondrial function and ROS production through positive feedback circuits involving p38MAPK, TGF-*β*, and mTOR, resulting in telomeric dysfunction and accumulation of senescent cells [108]. Furthermore, experiments in the presence of COX-2 inhibitors or specific silencing of p38MAPK demonstrated that increased subendothelial ROS concentrations activate p38MAPK, which activates p53 and the activating transcription factor-2 (ATF-2) and lead to COX-2 upregulation, explaining in part the appearance of senescence-related characteristics [109].

### 3.9. Lipid Oxidases

Lipoxygenases (LOX) are a family of enzymes that contain iron atoms at their center that oxidize polyunsaturated fatty acids, mainly arachidonic acid, to form a variety of hydroperoxides, forming as a by-product of the ROS reaction [110]. The ROS formation process occurs when Fe^3+^ present in activated LOX is reduced by the contact of this enzyme with its substrate [111]. The leading members of this family include 5-lipooxygenase (5-LOX), 12-lipoxygenase (12-LOX), and 15-lipoxygenase (15-LOX), and this classification is according to the carbon atom of arachidonic acid in which oxygen is inserted [111]. The main functions of LOX are to participate in the production of leukotrienes, prostaglandins, and thromboxane. They are also in charge of cell membrane lipid peroxidation [112]. The aging process is associated with an increase in LOX expression and activity, thus generating a significant increase in ROS and contributing to the effects of molecular inflammation related to aging [110]. This effect was evidenced with specific LOX inhibitors in aged animals, with an increase in LOX-dependent ROS generation associated with an increased expression of LOX mRNA [113]. The potent proatherogenic, proinflammatory, and pro-oxidant actions of leukotrienes are involved in vascular damage related to aging, such as vascular remodeling and oxidation of lipids from cell membranes [114]. Therefore, oxidative stress derived from LOX can be considered an important point in age-related inflammation.

## 4. The Exogenous Sources of ROS

There are several factors such as nutrients, alcohol, drugs (halothane, doxorubicin, and metronidazole), industrial solvents, heavy metals (Fe, Cu, Co, and Cr), transition metals (Cd, Hg, Pb, and As), air pollution, physical stressors (UV, X-rays, and among others) and lifestyle that act as exogenous sources of ROS [115]. Taken together, these stressors lead to oxidative stress and inflammation that directly impact human health, especially in the elderly.

Air pollution, for example, contains numerous toxic agents such as metals and other chemicals such ROS/RNS that drive the development of diseases. These harmful particles can be ingested as contaminants or incorporated by respiration into the organism, leading to local or systemic oxidative stress and inflammation [116]. In addition, chemotherapeutics and radiation can increase ROS and cause injury to the vascular, TGI, and hematopoietic systems, by enhancing oxidative damage in proteins, lipids, and DNA. In contrast, radiation-induced cell death can be mitigated or even prevented in mice with the antioxidant N-acetylcysteine (NAC) [117]. Similarly, cancer chemotherapy induces toxicity through oxidative damage. It is evident by the increased lipid peroxidation and reduced antioxidant and tissue GSH levels during chemotherapy [118]. Therefore, understanding the underlying pathways that lead to oxidative damage by exogenous sources and targeting by therapeutics imply preventing its deleterious effects.

## 5. The Antioxidant System

ROS concentrations are controlled and preserved in a live homeostatic system through enzymatic and nonenzymatic complexes of cellular detoxification, known as antioxidants [91]. Among the enzymatic system, superoxide dismutase (SOD), catalase (CAT), glutathione peroxidase (GPx), NADPH-quinone oxidoreductase-1 (NQO1), heme-oxygenase (HO-1), thioredoxin (Trx) ,and sulfiredoxins (Srx) are related to cellular defenses against oxidative stress [119]. Nonenzymatic antioxidants include low-molecular-weight compounds, such as vitamins (vitamins C and E), *β*-carotene, uric acid, and GSH, a tripeptide (l-*γ*-glutamyl-l-cysteinyl-l-glycine) that comprise a thiol (sulfhydryl) group [120].

### 5.1. Nonenzymatic System

GSH is highly abundant in all cell compartments, and the ratio of its oxidant/reduced form (GSH/GSSG) is the major thiol-based defense system against oxidative and electrophilic stress markers in the cell [121]. GSSG donates its electron to H_2_O_2_ to reduce it into H_2_O and O_2_. In addition, GSH also is a cofactor for several enzymes, such as GPx, which efficiently prevent oxidative insults and influencing together with glutaredoxin (Grx), the redox state of proteins via reversible S-glutathionylation [122, 123]. Indeed, post translational modifications such as cysteine oxidation via glutathionylation is coupled with GSH/GSSG, which reveals an important source of redox signals in cellular machinery.

*β*-carotenes are lipophilic compounds, members of the carotenoid family, found in various grains, fruits, oils, and vegetables and are precursors of vitamin A [124]. They present a robust antioxidant capacity that contributes to protecting the body against the effects of ROS. In addition, this compound is also related to increased levels of proteins related to GST (glutathione S-transferase), which plays an essential role against oxidative stress [125].

The structure of vitamin E is formed by four tocopherols (*α-*, *β*-, *γ*-, and *δ*-tocopherols) and four tocotrienols (*α*-, *β*-, *γ*-, and *δ*-tocotrienols) found in food [126]. Vitamin E is a major liposoluble, with antioxidant activity that scavenges peroxyl radicals by donating hydrogen from the phenolic group on the chromanol ring and terminates the oxidation of polyunsaturated fatty acids [126].

Vitamin C (ascorbic acid) is a hydrophilic molecule that can be found in its reduced or oxidized form [127]. Vitamin C has modulating effects. It is a potent antioxidant capable of preventing oxidative damage and lipid peroxidation induced by peroxide radicals, can reduce unstable biomolecules (nitrogen, oxygen, and sulfur radicals), and has the function of regenerating Vitamin E and other antioxidants in the organism [128].

Uric acid is the final product of purine metabolism on the oxidative activity of the enzyme xanthine oxidase in humans [129]. Uric acid plays a dual role in the redox biology context. At the first moment, it is an antioxidant that can remove ROS, such as singlet oxygen, and inhibits lipid peroxidation [130]. However, despite its antioxidant activity, high levels of uric acids can cause deleterious effects in the body, as exemplified by increased salt sensitivity and lipogenesis [131]. Furthermore, xanthine oxidase-related oxidative stress may also induce endothelial dysfunction and renal vasoconstriction [132].

### 5.2. The Enzymatic System

SOD is the major group of antioxidant enzymes that degrade superoxide anions [133, 134]. There are three isoforms of SOD in mammals: cytoplasmic Cu/ZnSOD (SOD1), mitochondrial MnSOD (SOD2), and extracellular Cu/ZnSOD (SOD3) [16]. All forms of SOD rapidly dismutase superoxide to the more stable ROS (H_2_O_2_), which is then converted to water and oxygen by either CAT or GPx [135]. Catalase is a highly efficient enzyme widely expressed in peroxisomes but may also be found in the cytosol or mitochondria. Its deficiency or malfunction is related to aging and age-related diseases [136]. GPx also catalyzes the reduction of H_2_O_2_ to water using GSH [121]. The catalytic mechanism of GPx involves the cyclic oxidation/reduction of catalytic cysteine or selenocysteine residues [137]. There are four isoforms of GPx that comprise GPx1, GPx2, GPx3, and GPx4, and the depletion of deficiency in one of these isoforms is related to the development of age-related diseases [138]. In addition, deficiency in GPx4 is implicated with ferroptosis, a nonapoptotic form of cell death, by increasing ROS, lipid peroxidation, and iron overload [139, 140].

Trx system comprises NADPH, thioredoxin reductase (TrxR), and Trx [141]. TrxR and GPx are dependent on selenium, which is often known as selenoproteins [142]. TrxR protects cells from oxidative stress through its disulfide reductase activity by regulating protein dithiol/disulfide balance [143]. In addition, the Trx system provides the electrons to thiol-dependent peroxidases to remove ROS with a fast reaction rate [144]. Trx antioxidant functions are also shown by involvement in DNA and protein repair by reducing ribonucleotide reductase and methionine sulfoxide reductases, plus regulating the activity of many redox-sensitive transcription factors [145].

NQO1 is a cytosolic antioxidant flavoprotein that catalyzes NADPH oxidation to NADP^+^ by various quinones [146]. NQO1 can reduce ubiquinone and vitamin E quinone to their antioxidant forms and reduce superoxide directly, suggesting a primary protective role [147]. It is highly inducible and plays multiple roles in cellular adaptation to stress [148].

HO-1 mediated numerous antioxidant activities, including in the cardiovascular system [149]. Activated by Nrf-2, HO-1, and its metabolites, including CO, Fe^2+^, and biliverdin, can prevent excessive oxidation of lipids and proteins by scavenging hydroxyl-free radicals, singlet oxygen, and superoxide anions and play an effective role in anti-inflammation, antioxidation, and antiapoptosis [150, 151].

### 5.3. The Antioxidant Transcription Factors

Nrf-2 (nuclear factor 2 related to erythroid factor 2), a transcription factor sensitive to redox potential, is the main regulator of the antioxidant system [152]. Under physiological conditions, Nrf-2 is downregulated in the cytoplasm by the Keap1 (Kelch-like ECH-associated protein-1) protein, leading to ubiquitination and consequent degradation via the proteasome which maintain a relative low Nrf-2 basal expression [153]. Under stress conditions, Nrf-2 dissociates from the complex and translocate to the nucleus, binding to the AREs (antioxidant response elements) and encoding the transcription of antioxidant enzymes and phase II detox, such as SOD, CAT, GPx, GR, HO-1, and NQO1 [154]. The cytoprotective effect of Nrf-2, at least in part, is related to its antioxidant properties [155]. Interestingly, ROS and other electrophiles are the primary activators of Nrf-2 by directly Keap1-cystine oxidation, leading to its dissociation and Nrf-2 translocation to the nucleus, which, in turn, will counteract ROS/RNS production [156, 157]. This process will adjust the redox potential at optimal level in healthy organisms.

In aging, the Nrf-2 is downregulated, being linked to cellular dysfunction, such as loss in proteostasis, genomic instability, and altered cellular communications [158]. In addition, a second mechanism that leads to Nrf-2 inhibition involves the Keap1 (cytosolic inhibitor), that can accumulate in the cytoplasm by different mechanisms such transcriptional regulation, epigenetic regulation, posttranslational modifications, and ubiquitination machinery dysfunction, resulting in low Nrf-2 activation, and consequently, the failure in transcription of antioxidant enzymes [159, 160]. Taken together, the mechanisms that lead to Nrf-2 downregulation is found in several age-diseases, such as CVDs, neurodegenerative diseases, and diabetes [161]. On the other hand, pharmacological and/or phytochemical that enhance Nrf-2 activation are very promisor target to prevent or treat age-related diseases [158, 162–164].

FOXO (Forkhead box O) is another transcription family of resistance factors with cytoprotective effects of controlling processes like energy production, oxidative stress, cell viability, and survival [165]. This group includes four members in humans (FOXO1, FOXO3, FOXO4, and FOXO6), being modulated by PI3K/AKT pathway [166, 167]. Thus, under oxidative stress conditions, overgrowth, or survival stimuli absence, FOXO proteins move to the nucleus, where their transcriptional functions can be performed [168]. FOXO3, a member of the FOXO family, has been associated with the regulation of oxidative stress, attenuating ROS through the transcriptional activation of SOD and CAT [169].

Oxidative stress controls FOXO upon c-Jun N-terminal kinase (JNK) activation and directly phosphorylates FOXO [167]. Furthermore, FOXO proteins promote numerous downstream target genes encoding antioxidant enzymes that regulate redox homeostasis, including superoxide dismutase (SOD) and catalase, in an NAD^+^-dependent manner [170]. In addition, genetic variants of FOXO3 are associated with longevity in worms, flies, and mammals, and protective alleles of FOXO3 are the second most replicated genetic factors related to prolonged human life, with findings of about 40 unique nucleotide polymorphisms (SNPs) noncoding consistently associated with longevity in humans [171].

Taken together, Nrf-2 and FOXO are the major transcription factors in cell protection [172]. Thereby, both are very promising pharmacological targets for age-related diseases.

## 6. ROS as Signaling Molecules

**Figure 2:**
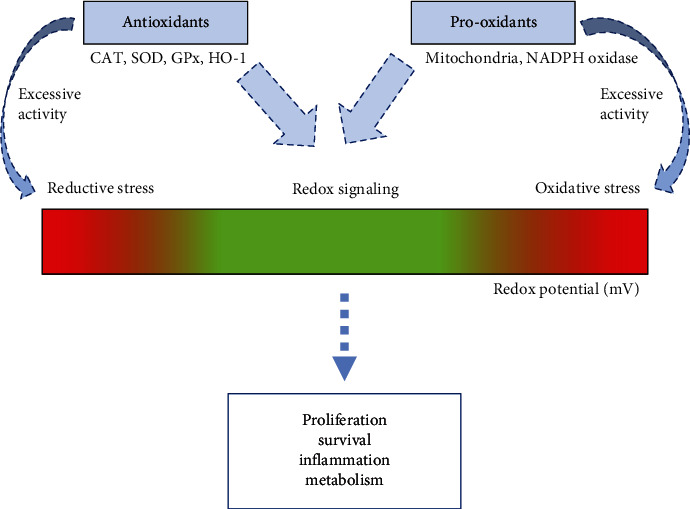
ROS at optimal level control cell fate by redox signaling. The balance of pro-oxidants and antioxidants maintains ROS at a safe zone for the cell. However, both the reductive (excess of antioxidant activities) and the oxidative stress can lead the cell to death. In addition, proliferation, survival, inflammation, and metabolism are some features controlled by redox signaling.

The oxygen molecule provided sustenance for aerobic life. ROS probably appeared on earth together with the first atmospheric oxygen about 2.4–3.8 billion years ago [173]. The biological systems have learned how to survive and created numerous mechanisms to live with these potentially harmful molecules [19]. On the other hand, the last decades of redox biology studies have shown ROS as signaling molecules [18, 174]. It has been described that ROS mediated several intracellular pathways, which include proliferation, inflammation, metabolism, and cell death (figure 2) [2, 19, 175]. The mechanisms behind cell fate depend mainly on the ROS concentration, compartmentalization, and time exposure [1]. Recently described redox-sensitive kinases and transcription factors that mediate redox signaling responses include Nrf-2, p38MAPK, HIF-, PGC-1, FOXO, and Nf-*κ*B [9, 175–177]. In fact, excess and persistent ROS lead to oxidative stress and are linked with several diseases. On the other hand, excessive reductive stress also has been linked with cancer, cardiomyopathy, and senescence [178, 179].

**Figure 3:**
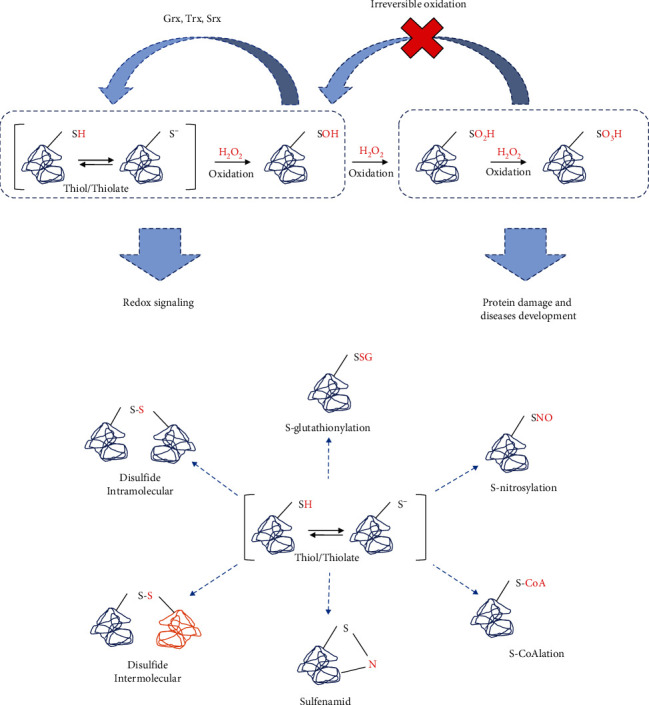
Cysteine oxidation is the site mainly described for redox signaling. Proteins sensitive to oxidation at thiols (cysteine amino acids contain thiols groups exposed for reaction) groups are a target for peroxide hydrogen and other oxidants. (a) First oxidation can change the conformation of the protein, which in turn will send a signal derived from this oxidation. Then, antioxidants can return the protein to its native form. However, in conditions such as oxidative stress, excessive oxidation can occur, driving proteins to irreversible inactivation, a process found in many age-related diseases. (b) Diverse oxidant can lead to a broad range of cysteine modifications that imply different signals relevant to cellular environment.

Cysteine oxidation is the main site of proteins on which ROS can act and induce signals by diverse range of posttranslational modifications [180]. The oxidant H_2_O_2_ interacts, for example, with Cys thiolate anions (Cys–S^−^) at physiological pH and oxidizes them to their sulfenic form (Cys–SOH), causing structural changes within the target protein and altering its function [180, 181]. Moreover, there is other reversible thiol modifications such as S-nitrosylation (Cys-SNO), S-glutathionylation (Cys-SSG), S-CoAlation (Cys-S-CoA), sulfenamid (Cys-S-N), and disulfide formation (Cys-S-S-Cys) that play a key role in redox signaling [177, 182]. These reactions are reversible, mainly by the activities of antioxidants such as Grx, Trx and Srx. However, failure in the antioxidant system may drive to persistent oxidation due to prolonged exposure to ROS leads to the irreversible oxidation status such as sulfinic acid (Cys-SO_2_H) and sulfonic acid (Cys-SO_3_H), leading to protein dysfunction, a key event in aging and age-related diseases [183] (Figure 3(b)). These redox-derived changes in protein function can affect transcription, phosphorylation, and other critical signaling events and/or alter metabolic fluxes and reactions in the cell by altering enzymatic properties. In addition, many redox relays exist in cells, and these can transduce and/or amplify an initial ROS-derived redox event [123, 177].

Moreover, recent evidence reveals other redox-sensitive sites apart from cysteine modifications, including protein carbonylation, methionine oxidation, and hydroxylation. For example, protein carbonylation occurs through direct oxidation of side chains of lysine, arginine, prolines, and threonine or by covalent attachment of products from lipid peroxidation (e.g., unsaturated aldehydes) [184]. Carbonylation is an irreversible protein modification that leads to protein inactivation that is unlikely to mediate physiological cellular signaling [185]. In addition, methionine also has sulfur atoms that may be oxidized similarly to cysteine; however, the reactivity of methionine oxidation is slower than cysteine oxidation, probably due to sulfur atom methionine space impediment [186]. In contrast, hydroxylation modifications may induce protein inactivation by directly interacting with valine, leucine, or lysine. Hence, increasing these amino acids' hydroxy forms is often related to aging-related diseases, such as atherosclerosis [187].

## 7. Impact of ROS on Biological Events in Aging

**Figure 4:**
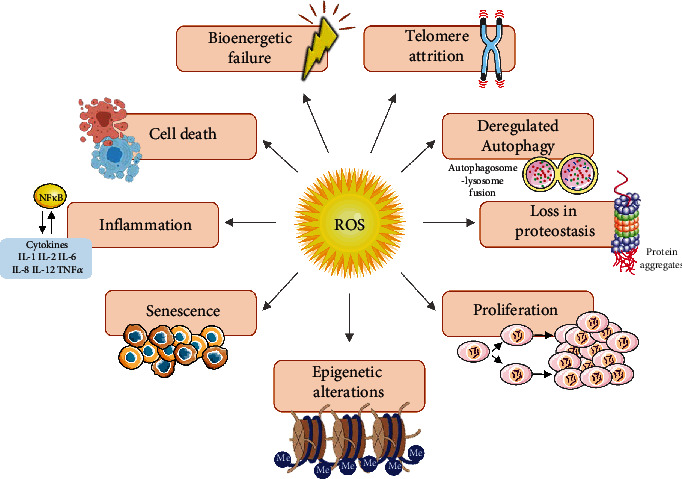
Crosstalk of ROS and the hallmarks of aging. The excessive production of ROS, a consequence of a redox imbalance, alters important physiological processes and increasing the susceptibility to numerous age-related diseases. Among those mechanisms include inflammation, cell death, bioenergetic failure, telomere attrition, deregulated autophagy, loss in proteostasis, proliferation, epigenetic alterations, and senescence.

The aging process involves numerous pathways, ranging from the molecular to the whole body. Although many theories try to explain these features, none of them appears to be entirely satisfactory. Connecting these theories may unify the aging process and help new targets for preventing or combating age-related diseases. ROS-mediated redox signaling is present in various biological events associated with aging, including inflammation, proliferation, cell death, senescence, autophagy, epigenetic alterations, proteasome dysfunction, telomere attrition, and dysregulated bioenergetics. In this context, ROS appear to be the major connector of cell fate in aging (Figure 4).

In addition, recent data support the tight connection between redox biology and the aging pathways. H_2_O_2_ is the most common type of ROS investigated, and these effects may differ in different types of ROS (See table 1).

### 7.1. Inflammation

Chronic inflammation “inflammaging” is one of the features of aging [197]. Oxidative stress and inflammation have many shared pathways and can be upregulated by vicious cycles [9, 198]. Overproduction of ROS is essential for the activation of AP-1 and Nf-*κ*B (redox-sensitive transcription factors) via kinases such as extracellular signal regulatory kinases (ERKs), c-jun N-terminal kinases (JNKs), p38 mitogen-activated protein kinase (p38 MAPK), protein kinase C (PKC), and phosphatidylinositol-4,5 bisphosphate 3-kin [199]. Moreover, ROS can directly oxidase I*κ*B at cysteine sites and induce the nuclear translocation of Nf-*κ*B for proinflammatory encoded genes [200]. Interestingly, many of the products encoded by NF-*κ*B are its activators, generating positive feedback, which means the more inflammatory mediators produced, the greater the expression of NF-*κ*B [201]. This cycle is accompanied by an increase in ROS levels, generating a stress environment related to the development of aging and age-related diseases [9, 202].

### 7.2. Proliferation

The rate of cellular proliferation is cell-type specific, being essential for growth, development, and tissue regeneration. The cell cycle is tightly controlled by a complex machinery of proteins such as cyclins and cyclin-dependent kinases (CDK) [203]. Moreover, cellular metabolism and its redox potential associated play a crucial role in cell cycle regulation. In mitotic cells, the progression of cell division is accompanied by an increase in ROS levels, especially in the mitochondria [204]. ROS influence cell cycle progression in a context-dependent manner via phosphorylation and ubiquitination of CDKs and cell cycle regulatory molecules [205].

Furthermore, ROS can activate growth factor receptors essential for metabolic needs in cell division [176]. It has been shown that ROS are able to control the G1 phase by ROS stimulating mitogenic pathways that control the activity of (CDKs) and phosphorylation of the retinoblastoma protein (pRB), thereby regulating S-phase entry [206]. However, an exacerbation of oxidative stress leads to cell cycle arrest [206].

Oxidative stress can injure DNA, leading to DNA damage response (DDR) [207]. DDR exerts checkpoint functions to block cell cycle progression and prevent the propagation of corrupted genetic information while damage is repaired [52]. Oxidative-induced DNA damage results in robust activation of the phosphatidylinositol-3-kinase (PI3K)/Akt pathway, including ATR, ATM, and DNA-PK [208]. The kinases are central components in DDR and act together with the DNA repair machinery to maintain cell genome integrity [209]. Interestingly, many proteins involved in DDR are endowed with a high number of cysteine residues as exemplified by Chk1, wee1 kinase, a specific CDK1 inhibitor, Chk2, Plk1 that allows cell cycle progression recovery after its arrest, and caspase 2, that is involved in apoptosis and is inhibited during G2 arrest by Chk1 [210]. These ROS-sensitive proteins undergo modifications in their structure and function through cysteine residue oxidation and disulfide generation depending on the cellular ROS levels [211]. If DDR cannot restore oxidative injuries in DNA, specific signals are generated to induce permanent cell cycle arrest (senescence) or cell death [212].

### 7.3. Telomere Dysfunction

Telomeres consist of thousands of nucleotide sequences at the end of each chromosome (5′-TTAGGG-3′, 9-15 kb, in humans) [213]. In somatic cells, after each cell division, part of these bases is lost in the process (~70 bp per year in humans) [214], promoting telomere shortening, a key hallmark of the aging process [4–6]. A shelterin complex composed of proteins and transcription factors is associated with telomeres [215]. This complex comprises a set of six subunits with distinct functions, which have essential participation in chromosome protection [216]. The telomeres participate in the maintenance of the genome and promote stability in the replication process, avoiding undesirable recombination and chromosomal fusion [217]. In contrast, telomere attrition is implicated in maladaptive cellular changes, cell cycle arrest, and diminishing tissue regeneration [213, 218]. Oxidative stress can induce telomere attrition through directly oxidative damage in DNA [219]. Oxidative DNA damage, particularly 8-oxoguanine (8-oxoG), represents the most frequent DNA damage in human cells, especially at the telomeric level [190]. Moreover, biochemical studies show TTAGGG repeats are preferred sites for iron-binding and iron-mediated Fenton reactions, which induce cleavage 5′ of GGG by hydroxyl radicals [220].

In some cellular lineages, such as stem cells, telomere shortening can be restored by the enzyme telomerase reverse transcriptase (TERT), together with its RNA component (TERC) [221]. The shelterin complex regulates both; however, excessive ROS is associated with downregulating TERT activity, TERC, and proteins of the shelterin complex, leading to massive cell death [222]. Taken together, these shreds of evidence reveal that oxidative damage shortens telomere length and is intrinsically related to the deleterious effect of these cellular events [220].

### 7.4. Epigenetic Alterations

Aging causes impairment of the epigenetic landscape and an increase in oxidative stress [223]. One of the mechanisms behind oxidative pathology is through epigenetic modifications, the three major types of epigenetic mechanisms: methylation/demethylation, acetylation/deacetylation, and histone modification [224]. DNA methylation was the first epigenetic modification discovered, and it is the best and most mechanistically understood [225]. The covalent addition of methyl (CH_3_) groups to DNA is controlled by DNA methyltransferases (DNMTs) [226]. In general, an increase in DNA methylation or hypermethylation of CpG islands at gene promoters is responsible for gene transcription suppression [227]. DNMTs can transfer methyl groups from S-adenosyl-L-methionine (SAM) and other methyl donors to DNA cytosine [226]. The enzymes responsible for maintaining the epigenetic status, such as DNMTs, histone methylase, and histone deacetylase (HDAC), could be affected by oxidative stress, causing a change in the methylation status of DNA [228]. However, alternative routes of DNA methylation changes at specific gene promoters may be independent of DNMTs, suggesting that superoxide can mediate nucleophilic substitutions, leading to changes in DNA methylation and histone modifications [229]. Superoxide neutralizes the positive charges of methyl donors, SAM, and acetyl-CoA, allowing them to deprotonate the cytosine molecule at the C-5 position and speed up the reaction of DNA with SAM resulting in DNA methylation [230]. Hence, these alterations may deeply impact aging [231].

### 7.5. Loss in Proteostasis

Protein homeostasis (proteostasis) is regulated by a complex machinery of regulators, such as chaperons, and as the main function of maintaining the quality control of proteins in systems biology, including protein synthesis, folding, conformational maintenance, and degradation [232]. In aging, proteostasis is deregulated, a key feature of aging and age-related diseases [233]. Oxidative stress contributes to proteostasis dysfunction in aging. In this context, oxidation can affect the process of protein synthesis and folding in the early stages, despite the mechanism of cellular stress adaptations such as chaperones [234]. In fact, unfolded protein conformations are the primary target for oxidative damage [235]. Moreover, ROS also can change native protein conformation mainly by cysteine and methionine oxidation, as previously mentioned (Figure 3), which leads to protein aggregates when persistent oxidation occurs, and, consequently, loss in function [234, 236]

Unfunctional proteins are degraded by two major proteolytic pathways, the ubiquitin-proteasome system (UPS) and the autophagosomal-lysosomal pathway (autophagy) [237]. In both proteolytic systems, molecular chaperones, such as heat-shock proteins (e.g., HSP70 and HSP90 families), are useful in recognizing misfolded protein species [238]. Chaperones assist proteins through the different conformational changes they undergo during their lifetime, including folding, assembly, disassembly, transport across membranes, and targeting for degradation [239]. In addition, chaperones target the unfolded protein for degradation via the proteasome or lysosomes [240].

### 7.6. Deregulated Autophagy

Cristian de Duve, who won the Nobel Prize in Physiology in 1974, discovered autophagy, a process of “self-eating” mediated by lysosomes in nutrient-depleted conditions [241]. Autophagy is a catabolic process essential for cellular homeostasis by removing cellular components, such as protein aggregates and damaged organelles [242]. Indeed, it helps maintain cellular energy levels during nutrient limitations through catabolic recycling processes [243]. Moreover, autophagy is deregulated or inoperative in aging, favoring “garbage” accumulation into the cell [244]. Although stress conditions such as glucose and amino acid deprivation are well-known inducers of autophagy, ROS and hypoxia have emerged as parallel regulators of autophagy machinery [245]. The interplay of autophagy and oxidative stress has gained notoriety recently. However, the mechanisms are still not fully understood. An important aspect that reveals a tight connection between them is that antioxidants inhibit autophagy [246]. In addition, studies using nutrient deprivation modeling have identified increases in O_2_^• -^ and H_2_O_2_ levels, which probably alter the cellular environment redox potential and interfere with the autophagy process [247].

Autophagy is controlled by the balance of two kinases: mammalian target of rapamycin (mTOR) and AMP-activated protein kinase (AMPK) [248]. The mTOR (autophagy inhibitor) is activated when nutrients are abundant [249]. In health, mTOR control a set of cellular events such protein synthesis, cell growth and survival [250]. On the other hand, AMPK is a sensor of cellular bioenergetics, such as the increased AMP/ATP ratio. AMPK is well known to inhibit mTOR, and also, pieces of evidence reveal that AMPK is directly activated by ROS [251]. In addition, sestrins protein family act as endogenous amino acid sensors, which regulate the balance of autophagy be stimulating AMPK and inhibiting mTOR [252]. Interestingly, sestrins protein family also activated by ROS. However, there is still debate if autophagy is upregulated to increase cell energetics or counter ROS production. In fact, damaged mitochondria (primary source of ROS) degradation is one of the critical roles of autophagy (mitophagy) [253]. p62 (autophagic machinery) has recently been implicated in the delivery of oxidized proteins to autophagosomes for degradation, which is most likely the mechanism by which oxidized proteins are eliminated [247]. Moreover, sestrins also binds to p62 and promotes the autophagic degradation, for example, of Keap1, thereby stimulating Nrf-2 (antioxidant responses) [254]. In addition, since sestrins regulates oxidative stress, autophagy, and numerous metabolic fluxes, its dysfunction contributes to aging and age-related diseases such cancer [252, 255, 256].

### 7.7. Bioenergetics

Cells developed specialized machinery that includes different sources and substrates to produce energy and sustain their metabolic needs. Most cells use glucose for ATP synthesis, but other fuel molecules are equally important for maintaining the cell energy status [257]. Although the oxidation pathways of fatty acids, amino acids, and glucose begin differently, these mechanisms ultimately converge into a common path, the TCA cycle, occurring within the mitochondria [258]. The intermediates enter the TCA cycle and give rise to the electrons donors, NADH, and FADH_2_. Hence, these reduced electron carriers are themselves oxidized via the electron transport chain (ETC), with concomitant consumption of oxygen and ATP synthesis [259]. In tumour cells, there is a shift in how energy is preference produced (glycolysis), probably an adjustment due to its high rate of growth and proliferation. In this case, the low number of ATP generated by glycolysis is compensated by pyruvate biomass, an effect called the “Warburg effect” [260]. In addition, autophagy can also produce cellular energy, especially in starvations conditions [261]. These processes have several regulators, such as insulin/IGF-1, mTOR, AMPK, sestrins and sirtuins, being implicated in aging and disease such cancer [4, 262].

In aging, there is a decrease in cellular energy supply, marked by the increased AMP/ATP and NADH/NAD^+^ ratios [263]. Indeed, mitochondrial dysfunction and deregulated autophagy and key features of the aging process, and oxidative stress plays a key role in this events [264]. In this context, ROS can, directly and indirectly, regulate energy status [265]. For example, ROS can activate AMPK, which activate a series of compensatory responses, including fatty acid oxidation (*β*-oxidation), inhibition of fatty acid synthesis, increased mitochondrial biogenesis, and stimulation of glucose uptake [266]. Moreover, mTOR inhibition (directly by ROS or via AMPK activation) leads to low protein synthesis and enhance autophagy, thereby decreasing anabolism, and consequently, increasing the resilience to ROS production [267]. However, in oxidative stress conditions, ROS also downregulate NAD^+^ levels through PARP activation, and consequently, inhibit sirtuins [268]. Sirtuins, known as genome guardian, control a set of transcriptions factors essential for mitochondrial health, antioxidants enzymes, proliferation, and metabolism [269–271]. The activations/inhibitions of multiple targets by ROS reveals its dual nature in health and diseases.

In contrast, a rise in intracellular NAD^+^ concentration following physical exercise, calorie restriction, and fasting activates SIRT1, which regulates several metabolic pathways by deacetylation of its target proteins [262, 272]. These proteins include histones, nuclear receptors, transcriptional coactivator: peroxisome proliferator-activated receptor-*γ* coactivator 1*α* (PGC-1*α*), Forkhead box (FOXO) transcription factors, and peroxisome proliferator-activated receptor-*α* (PPAR-*α*) [269]. Deacetylation of PGC-1*α* and FOXO transcription factors lead to enhanced mitochondrial respiration and catabolic breakdown of lipids, being essential for ATP production [257]. However, the role of ROS on cell bioenergetics is still unclear, which is usually attribute to ROS a dual effect depending on the context.

### 7.8. Cell death

Although an optimal level of ROS can promote survival, recent evidence indicates that redox modifications of proteins containing cysteine residues regulate multiple cell death modalities, including apoptosis, necroptosis, pyroptosis, and ferroptosis [145]. Redox reactions tight regulate these opposite effects, and the cell fate depends on the cellular environment's compartmentalization, time exposure, and concentration. In fact, ROS plays a vital role in controlling the cell cycle by activating, inhibiting, or even inducing protein gene expression. A dysfunction in this process leads to the activation of proteins that comprise the sophisticated machinery of cell death [273]. The most described pathway for cell death is apoptosis, which is regulated by numerous pathways dependent on redox modifications [274]. During mitochondrial dysfunction, several essential players of apoptosis, including procaspases, cytochrome C, apoptosis-inducing factor (AIF), and apoptotic protease-activating factor-1 (APAF-1), are released into the cytosol [275]. In addition, ROS reduce Bcl-2 (antiapoptotic) expression and induces Bax protein (proapoptotic), resulting in the downstream caspases leading to apoptotic cell death [276]. The Bax/Bcl-2 ratio is often used as an apoptotic marker in different cell types, including in aging studies [277]. In addition, ROS can increase Ca^2+^ in the mitochondria, altering the membrane potential and contributing to apoptosis [278]. ROS also can induce the expression of cell death receptors in the cell membrane, which include Fas (CD95/APO-1), Tnf-receptor 1 (Tnf-R1/p55/CD120a), Tnf-related apoptosis-inducing ligand receptor 1 [TRAIL-R1/death receptor 4 (DR4)], and receptor 2 (TRAIL-R2/DR5/APO-2/KILLER) [279]. On the other hand, antioxidants seem to delay the cell death process [280].

The p53 is a pleiotropic protein that regulates several pathways such as cell cycle, bioenergetics, metabolism, and apoptosis [281]. Moreover, ROS and p53 act together in controlling these processes [282]. However, more research is needed to clarify how ROS can control p53 action. It is well established that irreversible DNA damage by ROS evokes p53 and transcriptional activation as exemplified by downregulation of Bcl-2 expression and up-regulation of the Bax, fas, and Apaf1 expression [283]. Some researchers suggest that depending on the p53 levels, the effect might differ from survival, tumor suppression, or cell death [284].

### 7.9. Senescence

Cell cycle arrest characterizes senescent cells, mainly in the G1-phase, but the cells remain metabolically active [285]. Senescent cells secrete a variety of pro-inflammatory cytokines, interleukins, and growth factors, which has been reported as a “secretory phenotype associated with senescence” (SASP), which has an impact on itself as well as its neighbor environment [52]. Cells can undergo the senescent state (replicative senescence) after a certain number of cellular divisions “Hayflick limit” [286]. Moreover, cells can enter in senescent stage prematurely by several stressors such as oxidative stress, inflammation, cell energy starvation, and autophagy deficiency [4]. These events converge to DNA damage, epigenetic alterations, oncogenes activation, and chromatin changes [287]. Oxidative stress can induce DDR directly or indirectly by several pathways and, consequently, the activation of p53/p21 and p16/pRB pathways [288]. In both cases, they converge to inactivate the E2F family of transcription factors (essential for cell cycle progression) and, consequently, to senescence [289]. These pathways are tight connected to aging and age-related diseases. Although different senescence inducers exist, they all share the same aging phenotype, which includes increased cell loss, decreased antioxidant defense, and a decrease in bioenergetics. Thereby, senescence is a promising target for treating age-related diseases and improving lifespan and healthspan [290, 291].

## 8. Future Perspectives

In this review, we discuss basic concepts involving ROS and their implications on aging. Although its deleterious effects have been massively described, mainly when persistent oxidative stress occurs, in optimal levels, they are essential for cellular communication. However, grouping all ROS meaning similar function in the cellular context, implicate in the misunderstanding that all ROS must be downregulated at the same time. In fact, the difficulty in separating each ROS and their function as signaling molecules is still a challenge to be overcome. In addition, it is important to note that many oxidants will contribute to redox potential in the cell, not having necessary a target for signalization (e.g., cysteine oxidation). Thereby, the redox biology behind the activation and inhibition of numerous pathways may clarify the aging process and the implications on age-related diseases. The fact that different oxidants promote an increase in redox potential and this hallmark is involved in aging and age-related diseases makes us believe that an increase in cellular ROS is intentional by the cells to promote cellular survival mechanisms, requiring more and more ROS to have the same effect over time, a process that drives towards to the deleterious effects of ROS. In this perspective, understanding ROS as signaling molecules and considering their singularity, can lead scientists to pursue new pharmacological tools to control the main biological events determined by the redox potential, such as the hallmarks of aging, which implies an increase in the healthspan.

## Figures and Tables

**Table 1:** Interactions of H_2_O_2_ and aging pathways.

Mechanism	Target	Finding	Ref.
Inflammation	Nf-*κ*B/AP-1	H_2_O_2_-induced Nf-*κ*B/AP-1 phosphorylation and promoted cytokines releases such as Tnf-*α*, IL-6, and protein expression VCAM-1 and ICAM-1, plus increasing senescence markers such as *β*-galactosidase activity and *γ*H2AX, which result in HUVEC aged phenotype	[188]
Proliferation	Ki67/p16/p21/DNA	H_2_O_2_ showed a dramatic decrease in Ki67 expression, indicating cell cycle arrest, which was confirmed by an increase in subG1 phase and low S phase proportion, high p16 and p21 levels, and positivity for SA-*β*-Gal, indicating age phenotype in SH-SY5Y neuroblastoma cells	[189]
Telomere dysfunction	8-oxoG/TRF1/ TRF2	H_2_O_2_ induces 8-oxoG formation and reduces telomeric binding proteins TRF1 and TRF2 expression, leading to telomere shortening/dysfunction, which results in chromosome instability, especially end-to-end fusions, leading to senescence in fibroblast-derived from human fetal lung	[190]
Epigenetic alterations	Elovl2	H_2_O_2_ increases Elovl2 gene methylation, which disturbs lipid synthesis with increased endoplasmic reticulum stress and mitochondrial dysfunction, leading to metabolism dysfunction and senescence morphology, being correlated with human fibroblast aged phenotype	[191]
Loss in proteostasis	Cytosolic and nuclear proteins	H_2_O_2_ induced protein carbonylation, especially in the cytoplasm of young and senescent cells, whereas nuclear protein carbonylation was found only in senescent cells upon oxidative stress conditions, indicating that proteasomal systems can clear oxidized proteins in young but not in aged phenotype fibroblast cells	[192]
Deregulated autophagy	LC3/LC1/p62	H_2_O_2_ decreased autophagic flux by reducing the LC3/LC1 ratio and raising the p62 level, in addition to lowering pRb expression, which is essential to cell cycle progression, leading to HUVECs senescence	[193]
Bioenergetics	ETC/ATP	H_2_O_2_ promotes mitochondrial failure by decreasing mitochondrial membrane potential, oxygen consumption, ATP levels, and raising mitochondrial and cytosolic ROS in human neuroblastoma SH-SY5Y cell line	[194]
Cell death	Bax/Bcl-2	H_2_O_2_ reduces cell viability and increases the apoptotic rate by increasing Bax/Bcl-2 ratio, raising caspase levels, plus SA-*β*-Gal and inflammatory cytokines such as IL-6 and Tnf-*α*, leading to an aging phenotype in HUVECs	[195]
Senescence	*β*-galactosidase/p16/p21	H_2_O_2_ stimulate premature senescence by increase p16 and p21 expression which result in cell cycle arrest, in addition to enhance *β*-galactosidase activity in human skin fibroblast	[196]

Abbreviations: Nf-*κ*B (nuclear factor kappa B); AP-1 (activator protein 1); Tnf-*α* (tumour necrosis factor-alpha); IL-6 (interleukin 6); VCAM-1 (vascular cell adhesion molecule 1); ICAM-1 (intercellular adhesion molecule 1); *γ*H2AX (H2A histone family member X); HUVEC (human umbilical vein endothelial cells); Ki67 (marker Of proliferation Ki-67); SH-SY5Y (human neuroblastoma cell line SH-SY5Y); 8-oxoG (8-oxoguanine); TRF1 and TRF2 (telomere repeat binding factor 1 and 2); ELOVL (protein coding fatty acid elongase 2 gene); LC3 and LC1 (microtubule-associated protein light chain 3); ETC (electron transport chain); ATP (adenosine triphosphate); BAX (BCL2 associated X-apoptosis regulator); and Bcl-2 (BCL2-apoptosis regulator Bcl-2).
